# Immoralities in Church Management

**Published:** 1917-01-06

**Authors:** 


### IMMORALITIES IN CHURCH MANAGEMENT.

For over-thirty years, to our knowledge, the administrators of the Metropolitan Hospital Sunday Fund have been shocked and worried by the fact that in certain churches, irrespective of their denomination, the practice has prevailed to take from the offertories received on Hospital Sunday for the hospitals, a percentage, or a fixed sum, or some amount, which has been devoted to the payment of the expenses, or other matters connected with the place of worship in which the collection is held. Many times and oft has the problem been considered of how to awaken the moral conscience of the offenders in these cases, to the heinousness of their conduct, which disregards the purpose of Hospital Sunday in uniting on one day all religious denominations, to work for and give to the relief of the sick and suffering. For ourselves we lament the exhibition of incapacity made by the men who are responsible for these immoralities, which we regard as petty pilfering from sick funds to fulfil private business purposes. Such charges could have been more than adequately met, from other and proper sources, had these places of worship been blessed with a business management in control of its affairs.

1917 is undoubtedly the year of greatest opportunity for each individual, everywhere, throughout the nation, to awaken to a sense of his responsibility to his Creator for the blessings of health, for the mercies he has received, and for the protection he enjoys. Should not each man and woman be possessed by the determination, that, so far as they are concerned, nothing unworthy in act or thought shall emanate from them, as individuals, either in their business or in any other capacity of their lives, during 1917? 1917, in God's blessing, will prove a year of deliverance and freedom for the smaller nations, for the most defenceless members of humanity, for the re-establishment of Christ's Kingdom upon Earth, and for the awakening of the Allied nations and peoples to their dependence upon Almighty God, and to a realisation of the fact that they are at war in His cause and are fighting everywhere for the re-establishment and enforcement of the principles which Christ came and gave His Life to secure, for men and women. To recall the actual position of the Nation and Empire today, as each intelligent human being within its boundaries must see it, if he is worthy of the splendid opportunity which lies in front of him, is surely to make every reader feel that, the abomination of petty pilfering in connection with the Sunday Fund, and in connection with any other collection, must absolutely cease in every place of worship, within His Majesty's dominions, during the present year. We heartily pray that this indeed may be the case, but our knowledge of actual facts, at the moment, fills us with anxiety still.

On Sunday the 31st December, 1916, the last day of a memorable year, the day of the publication of the Allies' reply to Germany and the rejection of its sham offer for peace, was the day appointed for collections in every place of worship throughout the country on behalf of the Red Cross funds. It is incredible, but it is true, that any one of the myriad places of worship, where such collections were held, should through its minister or responsible managers, dare to receive in their collection plates or boxes money given by the congregations for the Red Cross, and to deduct from it a percentage, or worse, for "church expenses," or some other business expenditure connected with the particular conventicle involved. We can only say, that, at the church we attended with members of our family, all prepared and anxious to give our mite to the Red Cross collections, as an act of thanksgiving to Almighty God for the blessings of health, we found in the morning, it was announced from the chancel steps, that, such deductions would be made from the sum received for the Red Cross. This was bad enough, but at the evening service the practice followed was even worse. Then, in a voice which it was difficult to hear clearly, it was stated, as we understood, that the collection was for the Red Cross, *i.e.* that all the money given after the service would go to the Red Cross funds. It was further mentioned that boxes would be found at the door for further gifts to other funds, including church expenses. The alms of the congregation were given in good faith to the Red Cross, and some present who wished to contribute to church expenses also, on going out and examining the boxes at each of the doors of exit, found, that all the money given when the collection was made was presumably being taken for the purposes of the church, for there was no box for church expenses, but several boxes labelled Red Cross Fund. We consider that this grave evil, is a matter for the Archbishops, Bishops, and the Heads of other religious communities to take up promptly and exhaustively, that they may enforce such discipline or change of system, as to prevent the recurrence of a grievous and wanton scandal of this kind in the future. Steps ought to be taken to ascertain how much of the total sum received for the Red Cross in places of worship throughout the land on the last Sunday of the Old Year have been withheld under any such immoral systems or practices as those we have felt it our duty here to expose and condemn.

